# Glutaminase inhibition in multiple myeloma induces apoptosis *via* MYC degradation

**DOI:** 10.18632/oncotarget.20691

**Published:** 2017-08-24

**Authors:** Madlen Effenberger, Kathryn S. Bommert, Viktoria Kunz, Jessica Kruk, Ellen Leich, Martina Rudelius, Ralf Bargou, Kurt Bommert

**Affiliations:** ^1^ Comprehensive Cancer Center Mainfranken University Hospital Würzburg, Würzburg, Germany; ^2^ Institute of Pathology and Comprehensive Cancer Center Mainfranken University of Würzburg, Würzburg, Germany

**Keywords:** multiple myeloma, MYC, glutaminase

## Abstract

Multiple Myeloma (MM) is an incurable hematological malignancy affecting millions of people worldwide. As in all tumor cells both glucose and more recently glutamine have been identified as important for MM cellular metabolism, however there is some dispute as to the role of glutamine in MM cell survival. Here we show that the small molecule inhibitor compound 968 effectively inhibits glutaminase and that this inhibition induces apoptosis in both human multiple myeloma cell lines (HMCLs) and primary patient material. The HMCL U266 which does not express MYC was insensitive to both glutamine removal and compound 968, but ectopic expression of MYC imparted sensitivity. Finally, we show that glutamine depletion is reflected by rapid loss of MYC protein which is independent of *MYC* transcription and post translational modifications. However, MYC loss is dependent on proteasomal activity, and this loss was paralleled by an equally rapid induction of apoptosis. These findings are in contrast to those of glucose depletion which largely affected rates of proliferation in HMCLs, but had no effects on either MYC expression or viability. Therefore, inhibition of glutaminolysis is effective at inducing apoptosis and thus serves as a possible therapeutic target in MM.

## INTRODUCTION

The incurable hematopoietic disorder of terminally differentiated plasma cells—multiple myeloma (MM)—is believed to evolve through a multistep transformation process [1]. Progression of the disease is characterized by chromosomal abnormalities occurring as primary genetic events in the PC neoplasms as well as multiple late secondary alterations, including changes in the *MYC* locus (8q24) [2]. Recently, transcriptional activity of MYC has been described during late stages of MM progression [3]. In agreement with this we and others showed that MYC protein is expressed in primary MM patient material/samples, correlates with disease progression [4–6], and its expression is critical for the survival of MM cell lines [4, 7].

The underlying molecular mechanism of this MYC dependency in MM cells is unknown. In general, MYC assumes important regulatory functions in the cell cycle, proliferation, metabolism and apoptosis in all cells (for review [8]). Cellular metabolism encompasses among other processes both glycolysis and glutaminolysis. In normal cells, glycolysis largely generates pyruvate which is then fed into the Krebs cycle, and glutaminolysis breaks down glutamine (Gln) into alpha-ketoglutarate which is also a critical substrate in the Krebs cycle. However, the roles of these two metabolic processes change in tumor cells in that the pyruvate generated by glycolysis is converted to lactate (Warburg effect), and in the absence of pyruvate the Krebs cycle is fueled by alpha-ketoglutarate from glutaminolysis. Interestingly, MYC is known to influence both glycolysis and glutaminolysis by binding directly to the promoters of numerous genes involved in both pathways in transformed cells [9–12]. In some cell lines the restriction of glutamine is more potent in triggering cell death than glucose withdrawal [13, 14] and glutamine is crucial for the proliferation of cultured tumor cells [15]. Moreover, it has been reported that serum glutamine levels in MM patients are decreased relative to normal patients and patients in remission [16]. Taken together, these findings suggest that glutaminolysis is a worthy therapeutic target.

The first step in the glutaminolysis pathway is the transport of glutamine into the cells by the solute carrier proteins ASCT2 (gene symbol *SLC1A5*) and LAT1 (SLC7A5), before being catalyzed by glutaminases. Of the three known mammalian glutaminase isoforms, the expression of the GAC splice-variant (encoded by the *GLS1* gene) is modulated by MYC [12], GAC is also increased in some cancers and seems to be critical for the malignant cell’s transformation and survival [17–19]. The allosteric regulators BPTES and benzophenanthridinone 968 selectively inhibit the GLS1 derived proteins, interestingly however they do not affect non-transformed cells [18, 20, 21]. Therefore, we first tested whether glutaminase inhibition via BPTES or compound 968 is an attractive therapeutic target in MM, and then we asked what is the role of MYC in MM cell glutamine metabolism.

Here we show for the first time the induction of apoptosis by 968 mediated GLS1 inhibition in MM cell lines and primary patient samples. We show that indeed MM cell lines in culture are dependent on glutamine for survival and establish MYC’s role in this dependence. Importantly, we show for the first time that in MM cells MYC protein is degraded within minutes following glutamine-withdrawal and this degradation is a specific response to glutamine removal. Due to the current understanding of malignant metabolism and a lack of curative MM treatment, these promising results indicate that targeting MYC-dependent glutamine-metabolism in MM may provide a supplementary strategy for future therapies.

## RESULTS

### Glutaminolysis components in HMCLs and their inhibition by compound 968

**Figure 1:**
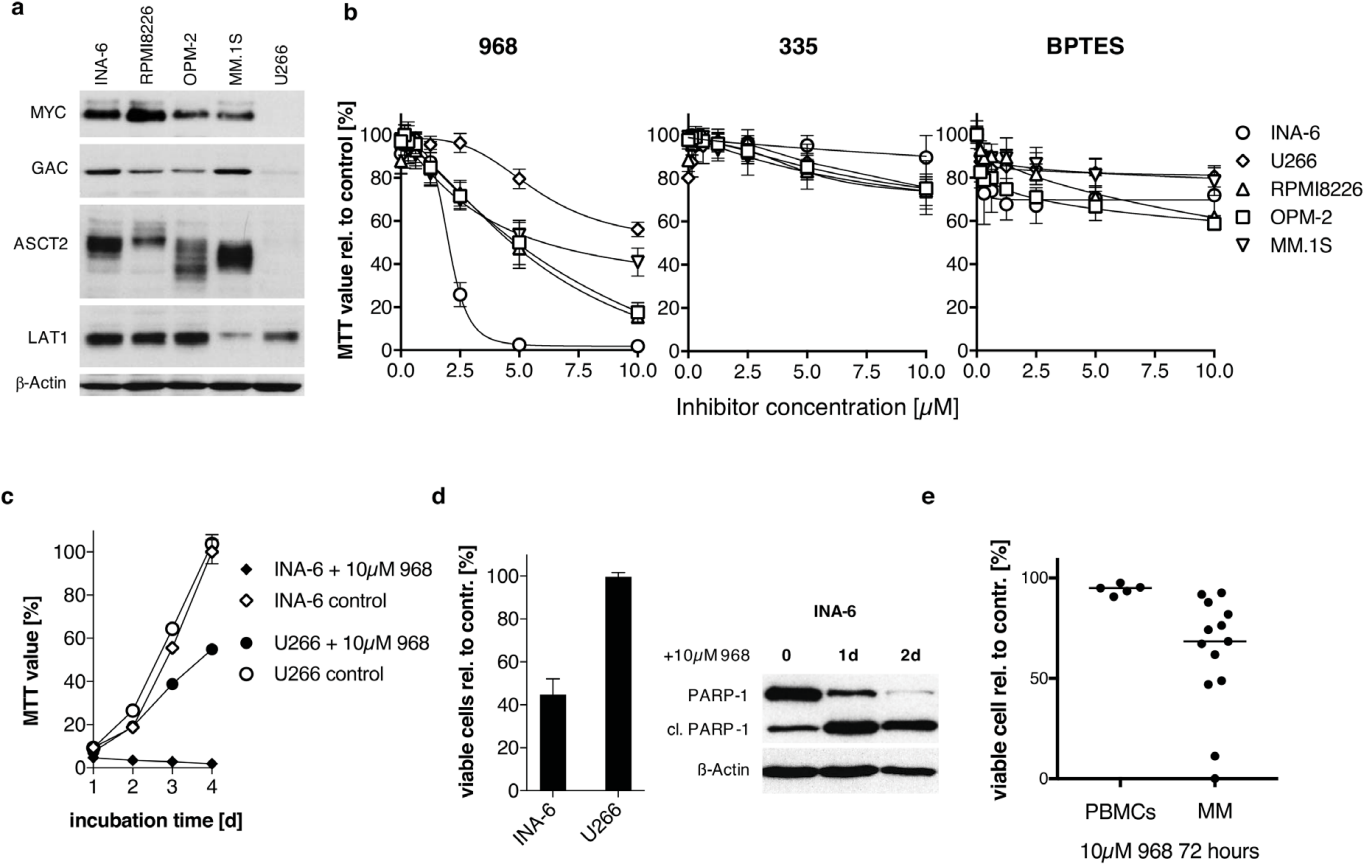
The effect of small molecule inhibitors on HMCLs and primary patient material **(a)** Western Blot analysis of MYC and selected candidates of the glutamine metabolism in untreated myeloma cell lines. All MYC-expressing cell lines (INA-6, RPMI-8226, OPM-2, MM.1S) show marked protein expression of glutaminase (GAC) and the substrate transporters (ASCT2, LAT1). U266 cells lack MYC protein expression along with the weakest levels of glutaminase isoform GAC and low of ASCT2 expression. **(b)** Growth-inhibitory effect targeting glutaminase. Enzyme inhibition was performed in a 2-fold serial dilution and measured after 5 days of incubation by MTT-assay using the small molecules 968 (n=3), its inactive control compound 335 (n=2) and BPTES (n=3). INA-6 cells are most susceptible to 968-treatment, whereas U266 cells show the weakest response to 10 μM 968 in relation to DMSO-control. BPTES reduced cell proliferation only moderately with a maximum of 40% independent of the treated cell line. **(c)** Comparison of INA-6 and U266 cell proliferation during 10 μM 968 treatment for 4 days analyzed by MTT. U266 proliferation was reduced by 50 % compared to DMSO-control on day four, while 968-treated INA-6 cells show no proliferation at all. **(d)** Treatment of INA-6 and U266 cells with 5μM 968 for 3 days reduced the percentage of viable Annexin V-FITC/PI negative cells in INA-6 by 50% whereas U266 cells showed no signs of apoptosis (n=3). Western Blot analysis on day 1 and 2 after 968 treatment confirms induction of apoptosis in INA-6 cells by PARP-1 cleavage (cl. PARP-1 indicates cleaved PARP-1 fragment). **(e)** Primary MM and peripheral blood mononuclear cells (PBMCs) were treated with 10μM 968 or DMSO as control for 72h and the percentage of viable cells (Annexin V-FITC negative, PI negative) compared to the DMSO treated sample were quantified using Annexin V-FITC and PI staining.

Expression of c-MYC (MYC) and selected candidates of the glutamine metabolism were examined in human myeloma cell lines (HMCLs). MYC protein is expressed in all tested HMCLs with the exception of U266, which is known to express L*-MYC* mRNA and protein but not c*-MYC* [25–27]. We used two different antibodies to identify glutaminase GLS1 splice variants (KGA and GAC) and found that KGA was evenly distributed between the cell lines (data not shown), whereas U266 had little GAC expression (Figure 1a). Glutamine substrate transporters LAT1 and ASCT2 was expressed to varying degrees in all tested HMCLs, U266 showed the lowest ASCT2 expression (Figure 1a).

Next, we studied the effect of glutaminase inhibition in HMCLs by the previously described small molecules compounds 968 and BPTES, and used the 968-structurally related, but inactive compound 335 as a negative control [18]. Compound 968 limited growth to varying degrees on the analyzed HMCLs, with IC50 values of 2.0μM ± 0.1μM for INA-6, 6.2μM ± 1.6μM for RMPI8226, 3.6μM ± 0.9μM for MM1.S, 9.0μM ± 4.5μM for OPM-2 and 5.7μM ± 0.56μM for U266 (Figure 1b). U266 cells were least sensitive as 10μM inhibited cell growth by 45% whereas 5μM fully inhibited INA-6 cell proliferation. Compound 335 showed little to no effect on the cell lines, with a maximal proliferation inhibition of 20%. BPTES-treatment also had limited impact (maximal 40%) on HMCL proliferation with 10μM BPTES.

Importantly, the findings of glutaminase inhibition by 968 are mirrored by the removal of glutamine from the medium (Supplementary Figure 1a). In particular, the removal of glutamine decreased the percent viable cells in INA-6 to 11.2% ± 4.2%, which was more sensitive than were U266 (89.3% ± 5.0%), U266-MYC (75.5% ± 5%), RPMI8226 (13.4% ± 4.2%), OPM-2 (28.5% ± 2.4%) and MM.1S (62.9% ± 1.3%). 10μM 968 inhibited growth of INA-6, where U266 proliferation is minimally affected (Figure 1c). Additionally, 968 induces apoptosis in INA-6 confirmed by activation of caspases using PARP-1 cleavage as reporter (Figure 1d). These data suggest that glutamine is critical for INA-6 proliferation likely through its interaction with glutaminase GAC and the substrate transporter ASCT2. In contrast, these proteins are both minimally expressed in U266 and likely reflect the resistance of U266 to glutaminase inhibition and glutamine loss.

Treatment of primary MM cells with 968 also induced apoptosis (Figure 1e). In 13 primary patient samples, there was on average a 62% decrease in viable cells in the presence of 10 μM 968 (p= 0.01 to DMSO treatment on non-normalized data). Treatment of five PBMC samples with 968 decreased the percentage of viable cells only to 94% compared to the DMSO control.

### The role of glutaminolysis in HMCLs survival

**Figure 2:**
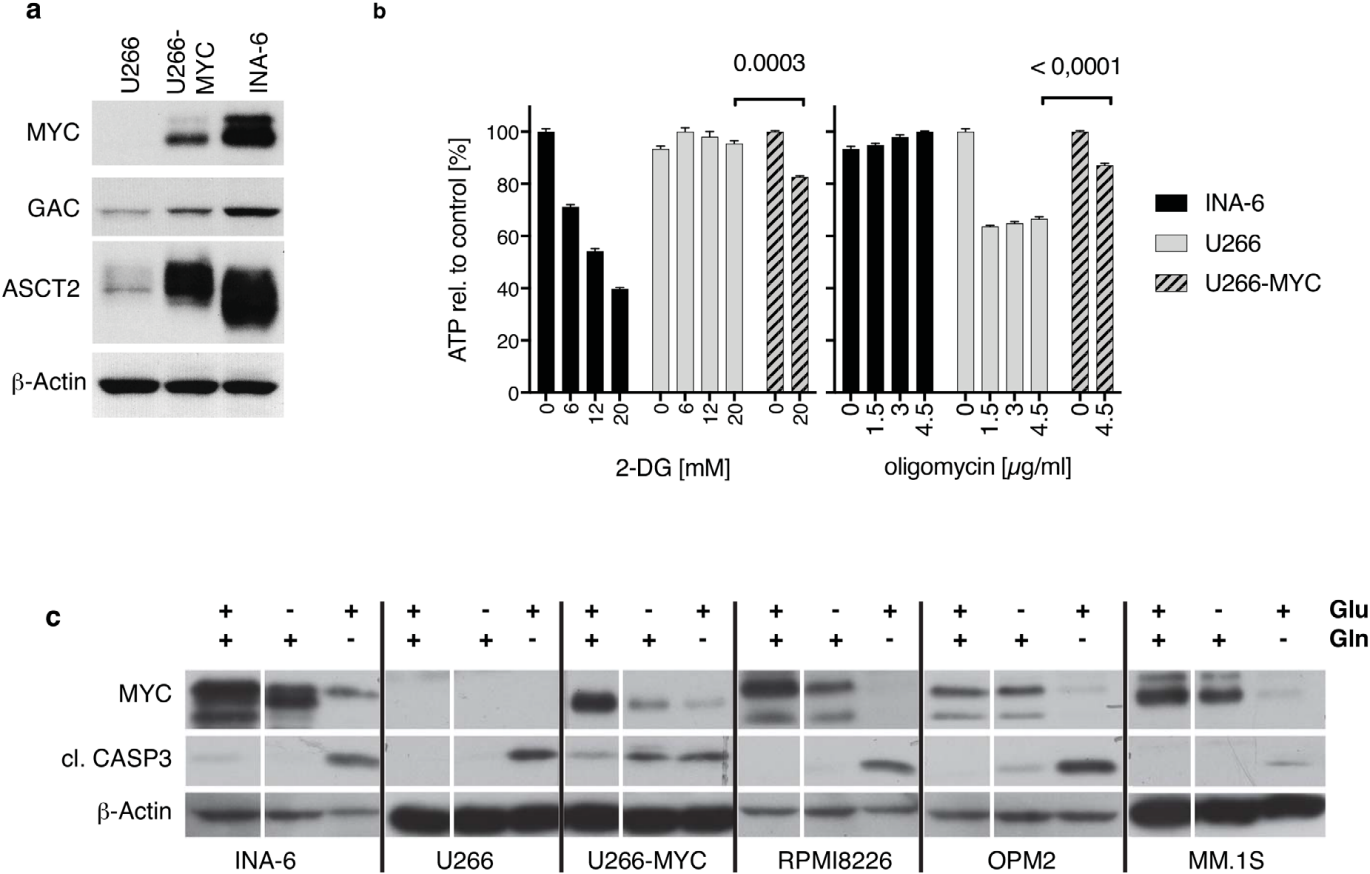
The role of MYC in glutaminolysis and glycolysis in HMCLs **(a)** Western Blot shows protein expression in U266 cells before and after lentiviral MYC-transduction (U266/U266-MYC) compared to endogenous expression levels in INA-6. MYC expression significantly elevates GAC and ASCT2 protein expression in U266 cells. **(b)** Oligomycin and 2-deoxyglucose (2-DG) treatment of INA-6, U266 and U266-MYC. Comparison of ATP-generation from glycolysis (2-DG incubation) and oxidative phosphorylation (oligomycin treatment) revealed that INA-6 cells synthesize ATP via glycolysis independent of oxidative phosphorylation as 2-DG reduces measurable ATP. In contrast, U266 produce ATP via mitochondrial respiration, which can be blocked using the ATP-synthase inhibitor oligomycin. INA-6 and U266 cells use contrary mechanisms to support ATP generation. U266-MYC cells are more sensitive to glycolysis inhibition and less sensitive to inhibition of the oxidative phosphorylation pathway (n=3). **(c)** Western Blot of MM cell lines incubated with or without glucose and L-glutamine in the culture medium for 24 hours. All HMCLs tested showed minimal changes in both MYC expression and generation of cleaved active caspase 3 when glucose is removed from the media. Removal of glutamine however diminishes MYC expression and increases cleaved active caspase 3 levels. For U266-MYC both withdrawal conditions reduce MYC protein while caspase 3 is cleaved.

To analyze the contribution of MYC to glutaminolysis and glycolysis in HMCLs we generated a MYC positive U266 cell line (U266-MYC) by lentiviral transduction. Transduced U266 cells were sorted using the co-expressed GFP. Figure 2a shows a western blot analysis of U266, U266-MYC and for comparison INA-6. When MYC is expressed in U266 there is an upregulation of the glutaminase isoform GAC whereas the KGA isoform expression levels are unchanged (data not shown), and the ASCT2 substrate transporter is strongly upregulated. Additionally, expression of MYC in the U266 cell line increased the cell proliferation rate from 2.9 ± 0.09 fold up to 4.4 ± 0.2 fold and cellular ATP content by 30 ± 4 % (Supplementary Figure 1b, 1c). Therefore, MYC expression induced transcription of proteins involved in both proliferation and glutaminolysis in U266. RNAseq analysis of the U266 and U266-MYC cell lines show that expression of MYC leads to upregulation of mRNAs coding for proteins involved in glycolysis and glutaminolysis. U266-MYC cells express more Hexokinase 2 (HK2) compared to U266 (log_2_ fold change +5.6). HK2 is a rate-limiting enzyme of the glucose metabolism pathway. Additionally, members of the solute carrier gene family, including SLC38A5 (log_2_ fold change +3.1) a glutamine and other amino acid transporter, SLC7A8, LAT2 (log_2_ fold change +4.8), SLC2A1 (log_2_ fold change +1.3) a glucose transporter, and SLC19A1 (log_2_ fold change +2.1) a folate transporter all have higher expression levels in U266-MYC (Supplementary Data File 1).

### MYC expression induces elements of both glycolysis and glutaminolysis in U266

It is known that some tumor cells expressing MYC are more dependent on glycolysis rather than glutaminolysis for ATP production [28]. INA-6 (MYC positive), U266 (MYC negative), and U266-MYC were used to test the contribution of each substance to ATP generation in these HMCLs. Figure 2b shows ATP levels over 30 minutes after either disruption of glycolysis using 2-Deoxy-D-glucose (2-DG) or oxidative phosphorylation inhibition with oligomycin at different concentrations. These data show that ATP levels in INA-6 cells are strongly inhibited by 2-DG in a concentration dependent manner (39.7% ± 1.0% at 20mM 2-DG), whereas ATP levels in U266 remains unchanged (95.5% ± 1.83% at 20 mM 2-DG) suggesting that glycolysis is critical for ATP production in INA-6. Oxidative phosphorylation is more important for ATP generation in U266 however, as its inhibition by 4.5 μg/ml oligomycin caused a decrease of 66.5% ± 1.6% in ATP but had no effect on the ATP levels in INA-6 (100% ± 0.4%). ATP levels in MYC expressing U266 cells are more sensitive to glycolysis inhibition by 2-DG (82.7% ± 0.6%) and less sensitive to inhibition of the oxidative phosphorylation pathway by oligomycin (87.1% ± 1.9%; Figure 2b). Nonetheless, ectopic expression of MYC in the U266 cell line decreased viability after glutamine removal (75.5% ± 4.3 viable cells; Supplementary Figure 1a).

In addition, we found that MYC expression was also strongly affected by removal of glutamine from the medium. Figure 2c shows that with the exception of U266-MYC, all HMCLs tested showed minimal changes in both MYC expression and generation of cleaved active caspase 3 when glucose is removed from the media. These data are in strong contrast to removal of glutamine from the media, where MYC level are diminished and cleaved active caspase 3 levels increases.

### Glutamine loss is reflected by rapid MYC degradation

**Figure 3:**
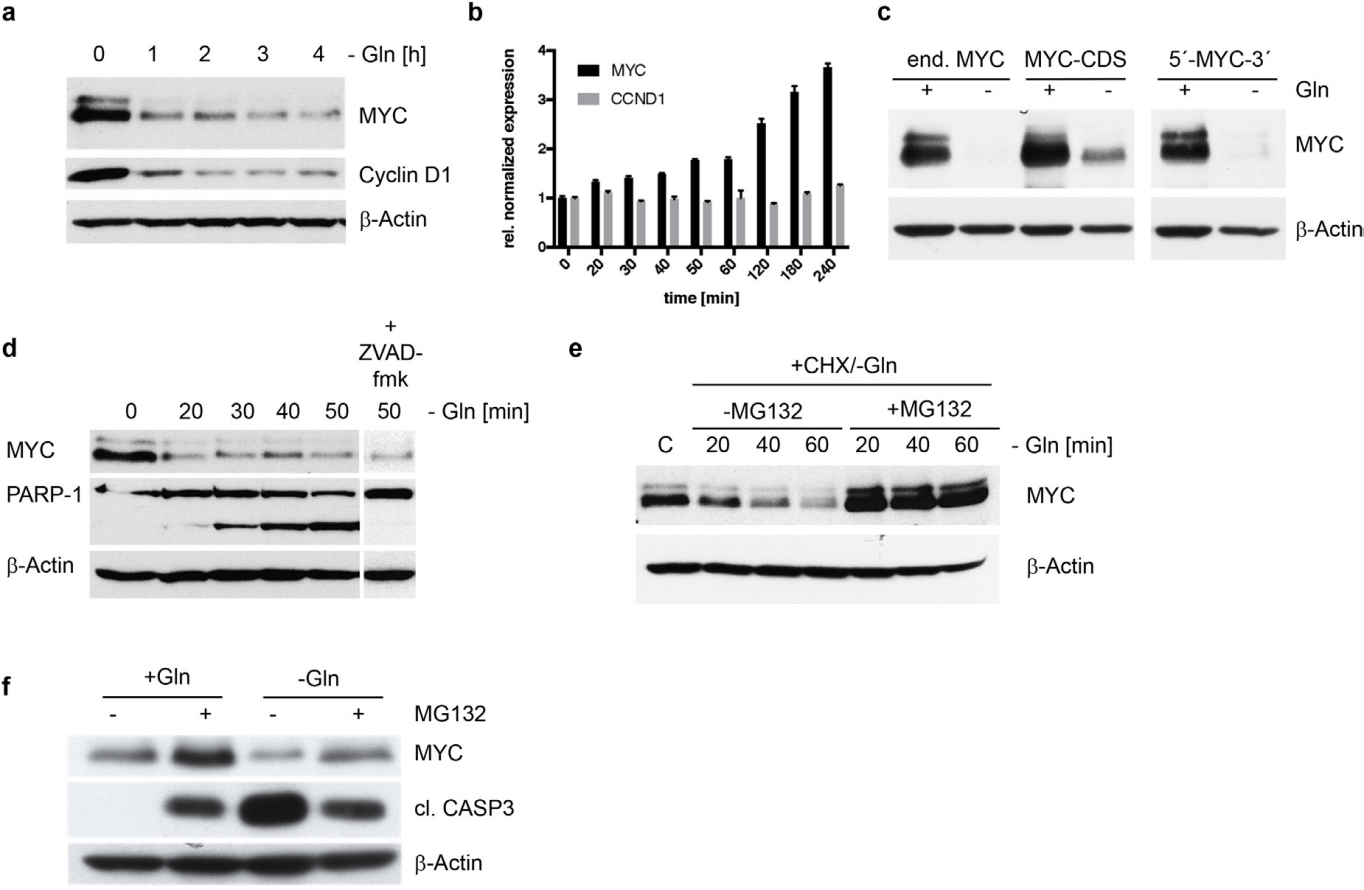
MYC degradation in INA-6 cells in the absence of glutamine **(a)** Western Blot analysis of MYC and Cyclin D1 expression in INA-6 following glutamine withdrawal over a time course of 4h. Protein levels are reduced significantly for both candidates already after 1h. **(b)** mRNA expression of MYC and CCND1 (Cyclin D1) using qPCR was measured after glutamine removal in INA-6. Relative to untreated cells MYC mRNA levels increase after 4 hours, while CCND1 mRNA levels remain unchanged under glutamine depletion. **(c)** Western Blot of INA-6 cells co-expressing MYC coding sequence (CDS) alone or MYC-CDS flanked by the 5’ and 3’ non-coding mRNA sequences together with endogenous MYC (end. MYC). Glutamine depletion induces MYC protein decrease independent of the expressed mRNA construct. **(d)** Time course of protein expression in INA-6 following glutamine removal shows rapid MYC degradation in concordance with increasing PARP-1 cleavage. Addition of a pan-caspase inhibitor ZVAD-fmk (10μM) did not prevent MYC degradation, but inhibits PARP-1 cleavage after 50 min of glutamine depletion. **(e)** Inhibition of the proteasome using MG132 (10μM) in the absence of glutamine stabilized MYC protein expression in INA-6 cells. Cycloheximide (10μg/ml) was added to control steady state protein levels by inhibiting mRNA translation. **(f)** Stabilization of MYC protein by MG132 mediated proteasome inhibition leads to reduced apoptosis after glutamine depletion. MG132 stabilizes MYC protein with and without glutamine. Stabilized MYC leads to reduced apoptosis after glutamine depletion as indicated by reduced levels of cleaved caspase 3 in INA-6 cells after glutamine removal and MG132 treatment.

We have demonstrated that MYC expressing HMCLs show a decrease in MYC expression and apoptosis in the absence of glutamine, next we analyzed the cause for decreased MYC expression. This decrease in expression can arise from either changes in transcription, translation or increased protein degradation. Within 1 hour of glutamine depletion in INA-6 cells there is a rapid decrease of MYC and Cyclin D1 protein expression (Figure 3a). A qPCR analysis for MYC and Cyclin D1 mRNA levels indicate that the loss of both proteins is not due inhibition of mRNA transcription (Figure 3b). In contrast the mRNA levels for MYC increase up to 3.7 ± 0.1 fold after 4 hours of glutamine depletion, while CCND1 mRNA levels remain stable.

To determine if miRNA mediated change in MYC mRNA translation efficacy was responsible for the decrease in MYC protein, we expressed different MYC constructs consisting of the MYC coding sequence alone or the MYC coding sequence flanked by the 5’ and 3’ non-coding mRNA sequences in INA-6 and U266 cells (data not shown). We found that glutamine depletion induces MYC protein decrease independent of the mRNA expression construct in both cell lines tested (Figure 3c). We have therefore excluded transcription and regulation of translation efficiencies as reasons for MYC depletion. Thus, we asked if MYC decrease was due to changes in protein degradation. In fact, MYC protein expression is already reduced within 20 minutes after glutamine removal, and accompanied by an equally fast PARP-1 cleavage (Figure 3d). The caspase inhibitor ZVAD-fmk does not prevent the reduction of MYC protein but clearly inhibits cleavage of PARP-1. These data indicate a post-translational regulation of MYC protein stability. We therefore asked if inhibition of proteasomal protein degradation by MG132 stabilizes MYC protein after glutamine depletion in the presence of cycloheximide. In INA-6 cells the proteasome inhibitor MG132 stabilizes MYC protein levels after glutamine depletion (Figure 3e). Furthermore, the therapeutically proteasome inhibitor a Bortezomib/Velcade displayed the same result (data not shown). These data demonstrate that glutamine depletion induces the loss of MYC protein by enhanced protein degradation. We then asked if pairing of both the degradation of MYC and induction of apoptosis in response to glutamine depletion were specific to glutamine withdrawal. To do this we first blocked the proteasome with MG132 and found that MG132 itself induces cleaved caspase 3 but no decrease in MYC protein as seen in the removal of glutamine from the medium. In the presence of MG132 however, glutamine removal induced less MYC degradation and cleaved caspase 3 (Figure 3f). Therefore, glutamine depletion induces the specific and coupled reaction of MYC degradation and the induction of apoptosis.

## DISCUSSION

Our data demonstrate that inhibition of glutaminolysis either pharmacologically, or by removal of glutamine from the medium leads to apoptosis in HMCLs. Of the two pharmacological blockers tested, compound 968 was more effective at inhibiting glutaminolysis. INA-6 was most sensitive to glutamine inhibition with 968 whereas U266 was least sensitive. With the exception of U266, inhibition of glutaminolysis induced growth inhibition in all HCMLs tested (Figure 1b). Furthermore, this induction of apoptosis was mirrored in experiments on primary patient material. Due to the small sample size, we could not establish a correlation between MYC mRNA or MYC protein level in the primary samples and the response to 968-mediated glutaminase inhibition. Nevertheless, our data demonstrate that targeting glutamine metabolism in MM could prove as a viable therapeutic target.

We have shown that inhibition of either glycolysis or glutaminolysis has different outcomes for HMCLs. Removal of glucose largely blocks proliferation of HMCLs, and inhibition of glutaminolysis induced apoptosis in most HMCLs. Similar findings have been documented in other malignant human cell lines [12, 14]. However, in another study on mostly non-overlapping group of HMCLs, it was noted that both glucose and glutamine removal induce apoptosis in all cell lines except U266 [29]. Clearly more studies in other laboratories are required to clarify this matter.

It could recently be demonstrated that HMCLs produce large amounts of ammonium in the presence of glutamine due to low expression level of glutamine synthetase. In addition, it could be shown that HMCLs and primary MM samples express high levels of the glutamine transporter ASCT2. Knockdown of ASCT2 induces apoptosis in HMCLs indicating a dependence of HMCLs on extracellular glutamine [30]. Interestingly the glutamine synthetase itself is under conditions of high glutamine levels targeted for degradation by the thalidomide receptor cereblon [31].

HMCLs express c-*MYC*, most notably excluding U266 which expresses L-*MYC* [25]. Both N- and c-MYC are known to drive elements involved in both glycolysis and glutaminolysis [8–10]. MYC drives glucose transporter expression and transcription of lactate dehydrogenase-A [28]. Additionally, MYC regulates the transcriptional program involved in glutaminolysis either by inducing expression of glutamine transporters [9] and/or via repression of glutaminolysis inhibitors [12]. Similarly, our data show that in HMCLs both of these metabolic processes are also influenced by ectopic MYC expression. U266 cells ectopically expressing MYC increased ATP production through glycolysis, and showed an increase in glutamine transporter expression (Figure 2A). Furthermore, we observed an enhanced expression of mRNAs coding for proteins involved in glycolysis, glutaminolysis and substrate transport. Similarly, the human Burkitt lymphoma P493-6 cell line increased the rate of glycolysis and glutaminolysis when MYC was conditionally over-expressed [32].

Most interestingly however is the novel finding that with the removal of glutamine from the media there was a rapid loss of MYC protein. In normal human fibroblasts, MYC expression is required for glutamine dependent apoptosis, but there was no change in MYC protein level after glutamine removal [14]. Here we show that the removal of glutamine from the media results in both rapid MYC protein degradation and induction of apoptosis in HMCLs. Remarkably, these events occur within 20 minutes of glutamine removal. We have previously published that siRNA mediated inhibition of MYC expression induces apoptosis in HMCLs, however this is a much slower process [4]. Here, the loss of MYC protein was shown to not be dependent on mRNA transcription as there was even an increase in MYC mRNA levels over the 4 hours studied. There was also no evidence of MYC mRNA translational changes as ectopic expression of MYC with 5’ and/or 3’ regulatory mRNA regions did not influence MYC degradation. However, when the proteasome is blocked, MYC protein is not degraded, demonstrating that the loss in MYC protein is due to proteasomal degradation. Finally, we showed that loss of MYC and induction of apoptosis occurred in parallel, and were specific to the inhibition of glutaminolysis.

These findings are interesting for many reasons. First, other studies on N-*MYC* in neuroblastoma or MYC in fibroblasts have not demonstrated MYC protein degradation [9, 33]. Therefore, it may be that this phenomenon is either specific to HMCLs or was simply not detected in studied samples. Second, we show that in all HMCLs MYC is degraded, but these HMCLs exhibit differing levels of viability. This is particularly interesting because MYC inhibition or loss is tied to apoptosis in HMCLs [4, 34], and thus there is an ongoing search for therapeutic agents targeting MYC. These data suggest that this strategy will not work if MYC is the sole target, and that a multiple target approach would be more effective at inducing apoptosis in MM cells.

Alternatively, it is possible that the loss of MYC protein induced by glutaminase inhibition would also have downstream effects that would render the cells more susceptible to the immune response *in vivo* because MYC regulates antitumor immune response through CD47 and PD-L1 [35]. Our data has laid the groundwork for these questions, by showing that HMCLs are indeed dependent on glutamine for their survival, that inhibition of glutaminolysis induces apoptosis in HMCLs, and finally that glutaminolysis inhibition leads to MYC protein degradation in all tested HMCLs.

## MATERIALS AND METHODS

### Cell culture and reagents

The human MM cell lines INA-6, MM.1S, RMPI8226, OPM-2 and U266 were maintained in RPMI-1640 medium containing 10% fetal bovine serum (FBS), 10mM HEPES, 1mM sodium pyruvate, 2mM L-glutamine, 2mg/l glucose, 100μg/ml Gentamycin (Biochrom). All components were purchased from Life Technologies, Darmstadt, Germany unless otherwise stated. INA-6 cells were supplemented with 2ng/ml human IL-6 (Biomol). INA-6 cells were a kind gift from M. Gramatzki, Kiel.

For experiments involving substrate deprivation, cells were cultured in RPMI lacking glucose/L-glutamine culture medium purchased from ThermoFischer Scientific (w/o L-glutamine) or c.c.pro GmbH (w/o glucose). Under L-glutamine-free conditions dialysed FBS (ThermoFischer Scientific) was used.

HEK293T cells cultured in 10% FBS DMEM supplemented with 10mM HEPES, 1mM sodium pyruvate, 2mM L-glutamine, 2mg/l glucose and penicillin/streptomycin. Glutaminase inhibitor 968 (AG-690/36107028) and inactive compound 335 (AG-205/05214045) were obtained from Specs and DMSO stock solutions were always sterile filtrated. BPTES was from Sigma (#SML0601). Oligomycin (Sigma #O4876), MG132 (Peptide Institute) and z-VAD-fmk (Bachem) were dissolved in DMSO, 2-Deoxy-D-Glucose in water (Sigma-Aldrich #D8375).

### Primary multiple myeloma cells and PBMCs

Primary Multiple Myeloma cells were obtained from diagnostic bone marrow aspirates of patients after informed consent with permission of the Ethics Committee of the University of Würzburg (76/13). The MM cells were purified from the bone marrow aspirate by magnetic CD138 MicroBeads and columns (Miltenyi) as described in [22]. Cells were cultivated in RPMI-1640, 10% FBS 10 mM HEPES, 1 mM sodium pyruvate, 2mM L-glutamine, 2 mg/l glucose, 100 μg/ml Gentamycin and supplemented with 2ng/ml IL-6. Peripheral blood mononuclear cells (PBMCs) were prepared from buffy coats using Ficoll density centrifugation.

### Western blot

Preparation of lysates is described in detail in [4]. Samples with 15μg protein/lane were separated by SDS-PAGE and blotted onto Immobilon-P PVDF membrane (Millipore). Membranes were blocked with 3% BSA in TBS-T (20mM Tris (pH 7.4), 0.5 M NaCl, 0.1% Tween-20). Primary antibodies (Abs) against MYC (clone Y69; Epitomics), GAC (#19958-1-AP; ProteinTech), ASCT2 (clone D7C12; Cell Signaling Technology (CST)), LAT1 (#5347; CST), Cyclin D1 (clone EPR2241; Epitomics), PARP-1 (clone F-2; Santa Cruz Biotechnology), ß-actin (clone I-19; Santa Cruz Biotechnology) were utilized. Antibody detection was achieved using horseradish peroxidase-conjugated secondary Abs and enhanced chemiluminescence substrate [23].

### Flow cytometric analysis

FACS-based cell counting was used to determine x-fold proliferation. Furthermore, fraction of apoptotic cells was identified after drug treatment by co-staining for 5 min with Annexin V-FITC (home-made) and 2 μg/ml propidium iodide (PI) or PI only in binding buffer (1x PBS with Ca^2+^ and Mg^2+^, 5mM EDTA, 0.5% BSA), washed with PBS and subjected to FACS analysis (CyFlow SL, Partec), FACS Calibur or LSRII both Becton Dickinson. Cells expressing GFP were analysed for apoptosis using the Zombie violet fixable viability kit (Biolegend) and annexin V-PE (ThermoFischer-Sientific) reagents.

### Analysis of glutaminase inhibitors via MTT assay

The Cell Proliferation Kit I (MTT) from Roche was used for quantitation of cell viability after glutaminase inhibition via 968, 335 or BPTES. Therefore, cells were seeded in 90 μl full medium in 96-well-plates. The respective inhibitor was pre-diluted in medium and reached the required concentration after adding 10 μl/well to the cell suspension. Control incubations with DMSO were always included. On the day of evaluation 10 μl MTT reagent was added for 4 h followed by 100 μl solubilization solution overnight at 37° C. Absorbance of the solubilized reduced formazan was measured at 570 nm with the microtiter plate (ELISA) reader (Sunrise, Tecan).

### ATP assay

To control for ATP production via glycolysis or oxidative phosphorylation each pathway was blocked separately using either 2-deoxyglucose or oligomycin and analysed using the CellTiter-Glo® Luminescent Cell Viability Assay (Promega). Cells were seeded at 100 μl in a white 96-well-plate and treated with the abovementioned compounds for 30 min. Cells were lysed directly in the plate by adding 100 μl CellTiter-Glo Reagent, incubated 10 min in the dark. The generated luminescent signal detected (MicroLumat LB96P, Berthold) is proportional to the amount of ATP in the cell.

### Lentivirus production

HEK293T cells were seeded at 300.000 cells/50ml/175cm^2^ five days before transfection. The medium was changed to 10% FCS DMEM w/o antibiotic 6 h before transfection. Prior to transection 2.5 ml OptiMEM were mixed with DNA (20 μg pHAGE-MYC constructs, 10 μg psPAX2 (Gag/Pol), 5 μg pMD2.G) and 2.5 ml OptiMEM with 140 μg PEI. PEI solution was mixed with DNA, incubated 15 min at room temperature and applied to the cells (psPAX2 and pMD2.G were a gift from Didier Trono (Addgene plasmid #12260 and #12259). After 6-8h medium was changed to 10% FCS DMEM supplemented with 1% sodium pyruvate, 1% Pen/Strep and 10 mM sodium butyrate. After 24 and 48h supernatant was collected, centrifuged at 300xg for 10 min and filtrated using PES-membrane (0.45 μm). Using a 20% sucrose cushion virus was concentrated via ultracentrifugation (2 h at 136.000xg). 50 μl of concentrated virus was used for transduction of HMCLs cells in 3 ml complete medium. Virus titer was tested on HEK293 cells and was between 1x and 5×10^8^ infectious particles per ml.

### Lentiviral transduction of c-MYC constructs

For stable MYC protein expression in U266 cells lentivirus was prepared from plasmids pHAGE-CDS-MYC and pHAGE-5’-MYC-3’, which express MYC coding sequence only or MYC-CDS flanked by 5’- and 3’-untranslated regions (UTRs) and GFP to allows cell sorting. MYC-CDS and 5´-MYC-3´plasmid were a kind gift from Martin Eilers, Würzburg. pHAGE-CMV-dsRed-UBC-GFP-W was a gift from Darrell Kotton (Addgene plasmid #24526) [24]. The day before transduction U266 were seeded at 250.000 cells/ml in 6-well plates. Following virus application plates were centrifuged at 1000 xg for 1 h in the presence of 4 μg/ml polybrene (Sigma-Aldrich), supplemented with fresh medium and incubated overnight. GFP positive cells were selected by cell sorting for GFP positive cells on a FACSAria III cell sorter Becton Dickinson.

### qPCR

cDNA was synthesized from 1 μg total RNA (NucleoSpin RNA isolation kit, Macherey-Nagel) using the High-Capacity cDNA Reverse Transcription Kit (ThermoFischer Scientific). Each cDNA sample was pre-diluted 1:20 and 4 μl were used with 4x Luminaris Color HiGreen qPCR Master Mix (ThermoFischer-Scientific) and 250 nM primer for the qPCR reaction.

The following primers were used: 5’-TGAGGAGACACCGCCCAC-3’ MYC forward, 5’-CAACARCGATTTCTTCCTCATCTTC-3’ MYC reverse, 5’-CCCTCGGTGTCCTACTTCAAATG-3’ CCND1 forward, 5’-TCTGTTCCTCGCAGACCTCCA-3’ CCND1 reverse, 5’-TGACTTTGTCACAGCCCAAGA-3’ B2M forward, 5’-CGGCATCTTCAAACCTCCAT-3’ B2M reverse, 5’-GGACGTTCAAGCAGATGGTTC-3’ MRPS14 forward, 5’-TCTTCACATCGCGCCACATT-3’ MRSP14 reverse, 5’-GCACAGAGCCTCGCCTTT-3’ ACTB forward, 5’-GAGCGCGGCGATATCATCA-3’ ACTB reverse. A two-step cycling protocol was performed as follows: UDG pre-treatment for 2 min (50 °C), initial denaturation 10 min (95°C), 40 cycles of 15 sec at 95°C (denaturation), 40 sec at 60°C (annealing/extension) (CFX Connect Thermal Cycler, Bio-Rad).

### Next generation sequencing

Quantitative transcriptome analysis using random-primed cDNA library from endogenous cell lines INA-6, U266 and the generated MYC-positive U266-CDS-MYC cells (GATC Biotech). Total RNA (100 ng/μl) (NucleoSpin RNA isolation kit, Macherey-Nagel) was used to generate a random-primed cDNA library. Isolation of poly(A)^+^-RNA, mRNA fragmentation, random-primed cDNA synthesis, adapter ligation and PCR amplification was performed by GATC. 5 million reads (1x 50 bp) of each sample were analyzed.

### Statistical and bioinformatics analysis

All data were analyzed with two-sided, unpaired t-tests and a P value <0.05 was considered significant. Values are expressed as the mean ± SEM of a minimum of triplicate, independent experiments. Inhibitor tests were analyzed using sigmoidal dose-response with variable slope using Prism 7.0. Annotations to pathways were performed using the MSigDB database from GSEA.

## SUPPLEMENTARY MATERIALS FIGURE




