# Supplementary material for: Glutaminase inhibition in multiple myeloma induces apoptosis via MYC degradation
Source: Oncotarget. 2017 Aug 24;8(49):85858–67. doi: 10.18632/oncotarget.20691 (PMC5689652; doi:10.18632/oncotarget.20691)
Supplement: Supplementary file 1 [file oncotarget-08-85858-s001.pdf]

Glutaminase inhibition in multiple myeloma induces apoptosis *via* MYC degradation

SUPPLEMENTARY MATERIALS

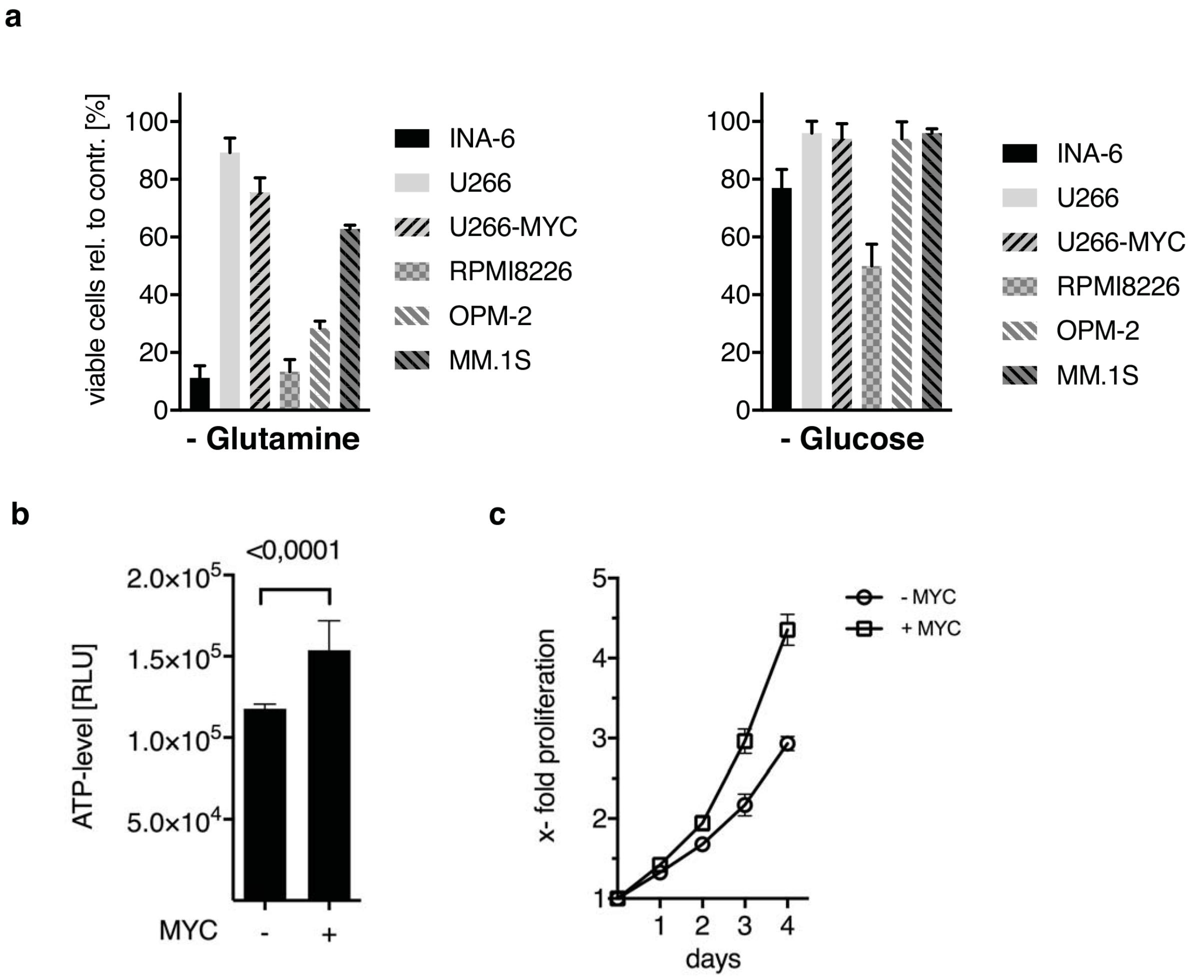

Supplementary Figure 1: (a) Induction of apoptosis measured by Annexin V/PI negativity after glutamine (left panel) and glucose (right panel) depletion after 3 days in HMCLs and U266-MYC cell line (n=3). **(b)** ATP-level of untreated U266 and MYC expressing U266 (U266-MYC) cells were measured using Promega's CellTiter-Glo Luminescent Cell Viability Assay (n=3). **(c)** U266 and U266-MYC cell proliferation was monitored on four consecutive days and blotted with regard to the seeded cell number at the beginning of the experiment (n=4).

Supplementary Data File 1: RNASeq data file, Excel containing all raw read counts from INA-6, U266 and U266-MYC as triplicate. Log2fold change is calculated for U266-MYC (divisor) compared to U266 (numerator)

See Supplementary File 1
